# A Case of Intussusception in a Patient with Marijuana Use: Coincidence or Possible Correlation?

**DOI:** 10.7759/cureus.7493

**Published:** 2020-03-31

**Authors:** Syeda Ramsha Zaidi, Zarak H Khan, Kashif Mukhtar, Munis M Ahmed, Sohaib Hasan Syed

**Affiliations:** 1 Internal Medicine, St. Mary Mercy Hospital, Livonia, USA; 2 Internal Medicine, King Edward Medical University, Lahore, PAK

**Keywords:** intussusception, marijuana, colonoscopy, intestinal obstruction

## Abstract

'Intussusception' means invaginating or telescoping and is caused by any condition that disrupts the normal physiological mechanism of intestinal peristalsis. Intussusception is rare in adults with an incidence of two to three cases per population of 1,000,000 annually. The most common cause of intussusception in adults is a neoplasm. In this case report, we are describing the case of a 22-year-old female with a past medical history of chronic constipation and a 60-pound unintentional weight loss who presented with the sudden onset of progressively worsening, severe abdominal pain associated with nausea, episodes of non-bloody, non-bilious emesis, and dark-colored loose stools. The patient’s social history was significant for extensive marijuana use for more than one year. Upon presentation, vitals were significant for mild bradycardia and examination was remarkable for diffuse abdominal pain. Initial laboratory testing was positive only for lactic acidosis. A computed tomography (CT) scan of the abdomen and pelvis revealed small bowel intussusception in the left hemiabdomen, along with periportal edema, and a small amount of pericholecystic fluid. The patient underwent both upper endoscopy and colonoscopy but no lead points for the intussusception could be identified. The patient responded to conservative management, including bowel rest, which resulted in the resolution of the intussusception on a follow-up small bowel series. Intraluminal irritants as the possible etiology of intussusception should be considered in the absence of a pathological lead point. Marijuana has been shown to act on various bowel segments and disrupts gastrointestinal motility through inhibition of cholinergic mechanisms. We believe the chronic use of marijuana could be the possible etiology of intussusception observed in our patient. Therefore, this case brings attention to the adverse effects of marijuana in light of increasing legalization and the increasing therapeutic use of marijuana and its derivatives.

## Introduction

John Hunter, who coined the term “introsusception” in 1789, was the first person to thoroughly describe the gross pathological features of this illness through his review of cases in pediatrics and adults [1]. “Intussusception” is described as the telescoping of a segment of the intestinal tract (the intussusceptum) into the lumen of the adjacent distal segment of the intestinal tract (the intussuscipiens). It is more common in the pediatric group, and adults represent only 5% of the total prevalence [2]. Frederick Treves classified the disease based on etiology in 1885 [3]. One cause could be idiopathic, which is benign and is more common among children. It is easily reducible using pneumatic enemas in nearly 80% of cases. Patients can also have a secondary etiology that can be a pathological lead point pulling in the primary segment or an intrinsic irritant, leading to irregular peristalsis. Ninety percent of cases among adults have a secondary cause, the most common of which are neoplasms, polyps, strictures, or diverticulum. Intussusception, in the absence of a lead point, has been reported mainly in patients with celiac or Crohn’s disease; however, it is mostly benign [1-2]. Intussusception accounts for 1% of all cases of intestinal obstruction in adults and has an annual incidence of two to three cases per one million [4].

The mechanism of intussusception is much similar to prolapse and involves imbalanced peristalsis that, with the support of a lead point, causes invagination of a bowel segment into the adjacent part. The intussusceptum can cause varying degrees of intestinal obstruction with acute total obstruction, mostly in children, that leads to the emergent acute abdomen in several cases. The intussuscepted segment, when pulled onto the mesentery, can compromise vascular flow, followed by ischemia (and even infarction) if not dealt with in a timely manner [4]. Diagnosis of the lead point using imaging or intraoperative findings becomes a necessity considering malignant neoplasms as possible underlying pathophysiology (approximately 30% of cases in adults) [4-5].

Although cannabinoids have shown strong non-inflammatory effects and positive results in preventing post-chemotherapy hyperemesis, paradoxical effects resulting in hyperemesis have been seen, specifically in chronic users as presented by Fernández-Atutxa et al. [6]. The pathogenesis is unconfirmed; however, studies have shown anti-peristaltic effects upon activation of cannabinoid receptor type 1 (CB1) receptors located in the submucosal and myenteric nerve plexus, as well as epithelial cells, throughout the gastrointestinal (GI) tract [7]. The alteration of GI peristalsis can cause intussusception as previously shown [8]. Herein, we present a case of chronic cannabis use as a potential cause of reversible intussusception, supporting its effects on the GI tract motility at a microscopic level. This case was also presented at the American Medical Association Research Symposium in October 2019 [9].

## Case presentation

**Figure 1:**
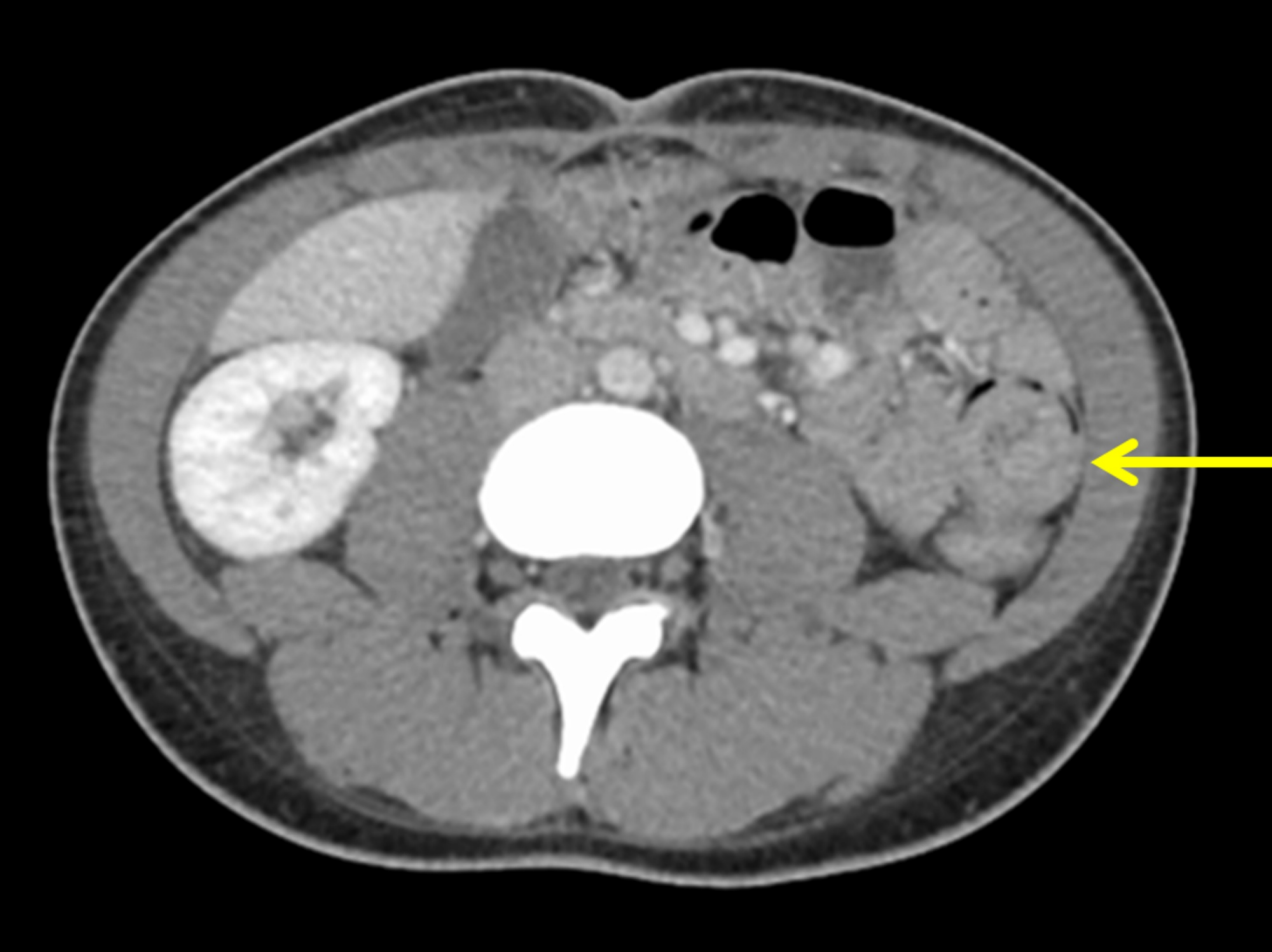
Computed tomography (CT) scan demonstrating intussusception in the left hemi-abdomen (yellow arrow) in our patient

A 22-year-old female, with a significant past medical history of chronic constipation for a year and 60 pounds unintentional weight loss over the last two years, presented with sudden onset of severe abdominal pain associated with nausea, vomiting, and diarrhea. Preceding these symptoms, the patient had a few episodes of abdominal pain associated with vomiting for two months that resolved on their own. She also endorsed having constipation for one year and had a bowel movement every four to five days. She had tried castor oil and probiotics with little relief. During this hospital admission, she had dark-colored loose stools for one day. She denied any frank red blood per rectum. She also had four episodes of non-bloody and non-bilious emesis. Of note, she had more than a one-year history of extensive marijuana use on a daily basis. She denied the use of over-the-counter non-steroidal anti-inflammatory drugs, dysphagia, regurgitation, heartburn, fever, chills, sick contacts, recent travel history, history of gallstones or appendicitis, dysuria, urinary frequency or urgency, and any gynecological problems. She was current with her vaccinations. Upon presentation, her vitals were unremarkable, except for mild bradycardia. Physical examination was pertinent for diffuse abdominal tenderness without guarding or rebound. Initial lab work, including complete blood count, electrolytes, hepatic panel, stool for occult blood testing, thyroid function tests, blood cultures, urine analysis, and human immunodeficiency virus (HIV) screening were unremarkable, except for an elevated lactic acid level (4.6 mmol/L, normal range: 0.67 - 1.8 mmol/L) which trended down to 1.2 later with intravenous (IV) fluid resuscitation. A pregnancy test was negative. A urine drug screen was positive for marijuana. Ultrasound of the abdomen showed trace pericholecystic fluid. She then underwent computed tomography (CT) scan of the abdomen and pelvis, which revealed a small bowel intussusception in the left hemi-abdomen, along with periportal edema, and a small amount of peri-cholecystic fluid likely representing ascites (Figure 1).

Gastroenterology and general surgery services were consulted. She was made nothing per oral (NPO) and subsequently underwent both an upper endoscopy and colonoscopy, which were normal. She was treated conservatively with bowel rest, antiemetics, and resuscitated with intravenous (IV) fluids. She gradually improved with the resolution of symptoms on Day 4 of her hospital stay. The patient’s intussusception had resolved on a follow-up small bowel series. She was discharged home with outpatient gastroenterology follow-up. 

## Discussion

Intussusception is a rare cause of intestinal obstruction that can result in secondary to several intrinsic factors affecting gastrointestinal motility [2]. Marijuana use as a cause of intussusception requires a high degree of suspicion since the presentation is similar to any other etiology as seen in our case study. Fernández-Atutxa et al. presented a case series comprised of three cases, each with a similar presentation and final diagnosis of intussusception without any identified organic lead points [6]. Chronic marijuana use was the only causative factor suspected in all three cases. Abstinence from marijuana resulted in an improvement in symptoms in one of these cases. Conservative management employed in two of the cases yielded promising results, similar to our case study [6].

Considering the fact that more than 90% of adult cases of intussusception are secondary to an organic pathology of which more than half are malignant tumorous lesions, it is imperative that patients undergo appropriate screening for diagnosis of etiology [4, 6]. Ultrasonography is a quick method of screening using findings, such as “target sign,” with a transversely oriented transducer or “pseudo kidney sign” when it is directed obliquely. However, the most sensitive modality with a diagnostic accuracy of 58% - 100% is using a CT scan, which has an added benefit of determining a neoplastic growth as the pathophysiologic factor. Noninvasive, as well as invasive modalities, such as barium studies, endoscopy, and colonoscopy, provide a detailed study of the GI tract and can prevent excessive operative management in the absence of warning signs [4-5]. Capsule endoscopy is a recent advancement among diagnostic modalities [10]. If, however, no organic lesion or malignant process could be identified preoperatively, conservative management has shown promising results.

Intraoperative management is employed in conditions of acute abdomen with the possibility of bowel infarction or perforation, or to biopsy or manage pathologic lead points of malignant origin. However, preoperative diagnostic modalities have reduced unnecessary surgical management and have improved outcomes through conservative management [4].

Patients with chronic marijuana use are at risk for developing intussusception, in the absence of a pathological lead point, due to a secondary alteration of GI motility. The major mechanism suspected is its receptor interaction in the GI tract, leading to inhibition of cholinergic neuronal pathways. Prokopchuk et al. presented a similar case with multiple intussusceptions in the intestinal tract and the only proposed causative factor was chronic cannabis use [8]. Adverse effects of prolonged marijuana use include addiction, substandard brain development causing cognitive impairment and psychosis disorders, chronic bronchitis, and others, most of which have a certain link to indirect cholinergic inhibition [11]. 

Epidemiological surveys have shown an increase in heavy marijuana use in people aged 12 or above, from 10.1% to 35.4% between 1992 and 2014, along with more than a 50% increase of marijuana-related emergency department visits within the United States [11]. The increased consumption of marijuana following its legalization and expanding therapeutic usage requires a detailed study of adverse outcomes. Our study aims to shed light on the causal relationship between the use of marijuana and intussusception, which can progress to fatal complications in certain cases.

## Conclusions

Extensive use of marijuana can lead to detrimental effects, such as intussusception, through its effects on GI motility, although the exact link between marijuana and intussusception has yet to be determined. A detailed diagnostic workup is required in adults to rule out other etiologies, and conservative management is the best approach. A recommendation to abstain from marijuana use can help in the prevention of relapse.

## References

[1] Stringer MD, Willetts IE (2000). John Hunter, Frederick Treves and intussusception. Ann R Coll Surg Engl.

[2] Weilbaecher D, Bolin JA, Hearn D, Ogden W 2nd (1971). Intussusception in adults: review of 160 patients. Am J Surg.

[3] Treves F (1885). The treatment of intussusception. Br Med J.

[4] Manouras A, Lagoudianakis EE, Dardamanis D (2007). Lipoma induced jejunojejunal intussusception. World J Gastroenterol.

[5] Warshauer DM, Lee JKT (1999). Adult intussusception detected at CT or MR imaging: clinical-imaging correlation. Radiology.

[6] Fernández-Atutxa A, de Castro L, Arévalo-Senra JA, Langara E, Cabriada-Nuño JL (2017). Cannabis intake and intussusception: an accidental association?. Rev Esp Enferm Dig.

[7] Izzo AA, Sharkey KA (2010). Cannabinoids and the gut: new developments and emerging concepts. Pharmacol Ther.

[8] Prokopchuk O, Neumann PA, Hüser N, Friess H, Wilhelm D (2019). Multiple intestinal intussusceptions caused by highly impaired gastrointestinal motility in a patient with chronic cannabis consumption. J Surg Case Rep.

[9] Khan Z, Paul J, Singh T (2019). Intussusception in a patient with marijuana use: coincidence or possible correlation?. Am J Gastroenterol.

[10] Culliford A, Daly J, Diamond B, Rubin M, Green PH (2005). The value of wireless capsule endoscopy in patients with complicated celiac disease. Gastrointest Endosc.

[11] Volkow ND, Baler RD, Compton WM, Weiss SR (2014). Adverse health effects of marijuana use. N Engl J Med.

